# Evaluating the effectiveness and acceptability of free door-to-door transport to increase the uptake of breast screening appointments in Yorkshire: a cluster randomised GP feasibility trial (DOORSTEP protocol)

**DOI:** 10.1136/bmjopen-2025-108616

**Published:** 2026-01-28

**Authors:** Mahboobeh Haji Sadeghi, Judith Cohen, Olufikayo Bamidele, Helen Roberts, Bronwen Williams, Beccy Acaster, Hannah Miles, Chao Huang, Lukas Pitel, Bryony Dawkins, Wessam Abass, Lesley Peacock, Una Macleod, Charlotte Kelly

**Affiliations:** 1 University of Hull, Hull, UK; 2 University of Leeds, Leeds, UK; 3 Hull University Teaching Hospitals NHS Trust, Hull, UK

**Keywords:** Early Detection of Cancer, Mass Screening, Breast imaging, General Practice

## Abstract

**Introduction:**

Breast screening uptake remains low in parts of the UK, partly due to barriers including limited transport access. Offering free transport to screening appointments may help address this and improve uptake. This general practitioner (GP) cluster-randomised feasibility trial will assess whether offering free door-to-door transport alongside routine screening invitations increases attendance.

**Methods and analysis:**

Eight general practices in Yorkshire will be randomised to either the intervention (routine invitation plus information about booking free door-to-door transport) or control (routine invitation only) group. Around 8000 women due for routine breast screening will be included. Primary feasibility outcomes include GP recruitment and randomisation, intervention fidelity, proportion of women from the 10% most deprived areas, acceptability and data transfer processes. Secondary outcomes include understanding travel behaviour, cost-effectiveness and screening uptake. Data will be collected from routine National Health Service (NHS) screening records, data linkage with NHS England, travel surveys and qualitative interviews exploring experiences and acceptability. Patient and public involvement is embedded throughout with members contributing to advisory and oversight roles.

**Ethics and dissemination:**

The trial has received ethical approval from the London–Harrow Research Ethics Committee, Section 251 approval from the Confidentiality Advisory Group and other relevant regulatory bodies. The University of Hull is the study sponsor. Results will be disseminated through peer-reviewed journal publications, conference presentations and plain English summaries for participants and the public. Findings will inform the feasibility and design of a potential larger trial to improve breast screening uptake via transport support.

**Trial registration number:**

ISRCTN17087898.

**STRENGTHS AND LIMITATIONS OF THIS STUDY**

- Robust study design that will produce data to support regulatory approval.
- The statistical analysis methods are appropriately chosen, ensuring reliable and valid results.
- It may not be possible to interview a sufficient number of women who did not attend the screening due to a lack of interest in participation.

## Introduction

 Breast cancer is the most commonly diagnosed cancer among women in the UK and in Yorkshire alone around 4300 women are diagnosed with breast cancer each year.^1^ Survival is closely linked to stage at diagnosis: 5-year survival for women diagnosed at Stage I is 97.9%; but falls sharply to 26% for those diagnosed at Stage IV.^2^ The National Health Service (NHS) breast screening programme was introduced to promote earlier detection, thereby improving survival outcomes.^3^ All women aged 50–70 years old are invited for breast screening every 3 years, yet uptake remains suboptimal. Yorkshire is a large region in northern England, comprising urban and rural areas with a diverse population. In Yorkshire, only 71.4% of eligible women attended their most recent screening appointment, with significant variation across the region.^4^ National data indicate that on average, 8.4 breast cancers are detected per 1000 women screened.^5^ Based on this rate, approximately 197 000 women did not attend screening in Yorkshire over a 3-year period, representing an estimated 1655 potentially detectable cases of breast cancer that may have been diagnosed at an earlier stage.^4 5^ Public Health England identifies 70% attendance as an acceptable threshold, but 80% as achievable.^6^ While Yorkshire marginally exceeds the acceptable level, it falls short of the achievable benchmark, with notable disparities in screening uptake across the region. Addressing barriers to participation is therefore a public health priority, particularly in areas of high deprivation and poor access to services. Improving uptake has the potential to significantly impact early detection and survival outcomes across the region.

Non-attendance at breast screening appointments is influenced by a range of factors, including a perceived lack of personal risk, cultural and religious beliefs, previous negative experiences, language barriers, embarrassment and practical difficulties in accessing the screening site.^7^,^9^ One of the most commonly reported barriers is limited access to screening facilities—either directly (eg, due to transport costs) or indirectly (eg, competing time commitments). Several studies have found that women who live further away from breast screening sites are significantly less likely to attend.^10 11^

One possible approach for increasing screening uptake rates is to target interventions that ensure women can get to breast screening facilities ‘…at reasonable cost, in reasonable time and with reasonable ease’.(p6)^12^ The introduction of mobile breast screening vans targeting reducing the distance needed to travel by bringing breast screening facilities to local communities has been one potential solution to this issue.^10^ However, constraints on where they can be located due to the van size, need for access to electricity and parking for operational staff and attendees have resulted in them not always being located in sites that are easy to get to without a car, or are based in areas unfamiliar to the women who need to attend.^7^ Additional measures such as providing transport to reduce the physical barrier between home and screening site are still needed to allow some women to attend their appointment.

Providing free door-to-door transport to support access to healthcare appointments for individuals facing mobility or logistical challenges is not a new concept. The Yorkshire Ambulance Service alone conducts approximately one million non-emergency journeys each year, transporting patients to hospital appointments.^13^ However, women invited for routine breast screening are not eligible for this transport provision.^14^ Several studies have explored interventions to improve access to screening services, including the use of taxis, bus passes and transport vouchers.^15^,^17^ For example, Bell *et al*^16^ implemented a multicomponent intervention in three inner-city Cardiff general practitioner (GP) practices, which included the offer of transport to the screening centre. The intervention was associated with a 15% increase in attendance compared with previous screening rates within those practices. However, this study was not a randomised controlled trial, lacked a comparison group and did not isolate the effect of transport from other components of the intervention package. We hypothesise that offering a free, convenient, door-to-door transport service will improve breast screening attendance in Yorkshire, particularly among underserved populations. At present, there is insufficient evidence to inform commissioning decisions regarding transport support for breast screening attendance. A large-scale trial would be required to provide robust evidence of effectiveness and cost-effectiveness and subsequent commissioning of door-to-door transport. To address some of the uncertainties about the delivery of a definitive trial, we are undertaking a feasibility trial. This trial aims to assess the acceptability, fidelity and potential impact of a dedicated transport intervention on breast screening uptake.

## Methods and analysis

This protocol was prepared in accordance with the SPIRIT (Standard Protocol Items: Recommendations for Interventional Trials) 2013 reporting guidelines.^18^

### Study aims and objectives

This feasibility trial is a necessary first step to evaluate the practicality of trial procedures, the fidelity of intervention delivery and to provide preliminary signals of effectiveness, estimate cost effectiveness and generate data to inform sample size calculations for a future definitive phase III randomised controlled trial.

The primary aim of this study is to assess feasibility across key domains:

- Recruitment of GP practices.
- Implementation of the intervention.
- Data collection processes.
- Acceptability of the intervention.

In addition, the study will:

- Provide an initial indication of efficacy.
- Generate preliminary cost-effectiveness data.
- Inform the design and sample size calculations for a potential definitive phase III randomised controlled trial.

Screening attendance data will be obtained from the NHS Breast Screening Service (NHS BSS) and this will be linked via NHS England through the Data Access Request Service (DARS) to deprivation and ethnicity data.

Travel surveys will be administered to participants in both the intervention and control arms to explore how women travel to screening appointments. To further assess the acceptability of the intervention, a subset of women from the intervention group—as well as representatives from the transport provider—will be invited to participate in qualitative interviews. These interviews will explore experiences of the transport offer, barriers or facilitators to use and perceptions of acceptability.

### Study design

The intervention is the offer of free door-to-door transport to a routine breast screening appointment for all eligible women registered at GP practices randomised to the intervention arm of the trial. All women registered with GP practices in the intervention arm, who are due to be invited to routine breast cancer screening within the intervention time window, will be given information on how to book transport to their appointment. The information will be delivered through a letter (detailing the offer) that is included at the same time as the routine screening invite letter sent by the Humber and East Yorkshire (HEY) BSS. The transport offer will include being driven from home (or workplace) to the appointment; the driver will wait for the appointment to finish and drive the participant back. Taxi companies based in the two areas of East Riding of Yorkshire (ERY) and Kingston-Upon-Hull (Hull) will be used. The transport provider will provide transportation services, including any drivers licensed to carry out private hire bookings in accordance with the private hire vehicle regulations. The trial will cover the costs of the journeys.

The free transport will be offered with the first HEY BSS screening invitation letter sent to the women at an intervention GP. If the women do not attend the first appointment offered, then a second invitation will be sent routinely from the screening service and free transport will again be offered at this stage. If the women do not attend the second appointment offered by the HEY BSS, then no further appointment will be sent by the screening service. The women can then use the free transport up to 6 months from the first invitation letter if they subsequently book the screening appointment themselves.

If any of the participants in the intervention arm need a recall for a further screening assessment, they can also still use the free transport offer.

**Figure 1:**
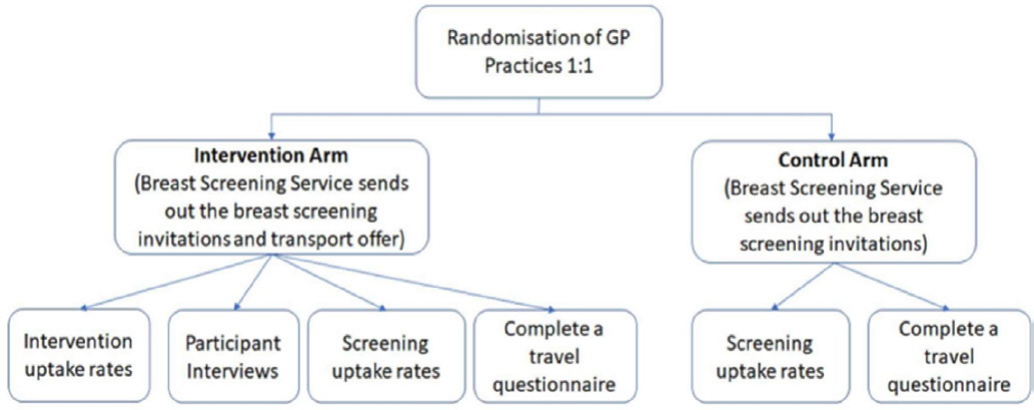
Recruitment randomisation, participant flows and data collection. GP, general practitioner.

Figure 1 outlines recruitment, randomisation, participant flows and data collection (see separate document uploaded for Recruitment, randomisation, participant flows and data collection).

### Study population

The study is a cluster randomised feasibility trial, with clustering at the level of the GP practice. Eight GP practices will be included in this study. GPs will be allocated to the intervention group or the control group. The flow of participants is shown in figure 1 (see separate document uploaded for Recruitment, randomisation, participant flows and data collection). A GP cluster randomised design has been proposed instead of individual patient level randomisation for two reasons: the complexity of individual randomisation and preparation of letters with/without transport information for the screening service; and the letter inviting women to book transport may be shared or discussed between women who are registered at the same practice or live in the same area, leading to trial contamination. Known issues of requiring larger sample sizes and recruitment bias in cluster randomised controlled trials (RCTs) are mitigated as all women at intervention practices will receive the intervention letter and anonymised routinely collected outcome data are being obtained.^19^

Women at participating GP practices will receive the standard screening invitation letter at the usual planned time from the screening service, with women registered at an intervention GP practice receiving additional information about the transport booking instructions. A participant notification poster will be displayed at GP practices during the trial.

We estimated 8000 participants will be included in the trial based on the number of eligible women (between ages 50 and 70) at eight average sized GP practices. This estimate was derived from internal data provided by the local BSS in which the study is being conducted. The sample size of eight practices will allow for the reliable estimation of key feasibility figures and detection of signals of efficacy for the future full trial.^20^

### Study recruitment

The eight GP practices from ERY and Hull will be identified with the support of the local Clinical Research Network (CRN) and the HEY BSS schedules. The schedule for inviting women for breast cancer screening from GP practices is determined 3 years in advance, running on a 3-yearly rota. GP practices whose registered women are scheduled to be invited in the 12-month intervention phase of the study will be selected to participate. All women invited for a routine breast screening appointment who are registered at the selected practices will be included in the trial. The HEY BSS has one permanent screening site at Castle Hill Hospital and two mobile vans that serve ERY and Hull. Women are invited to attend the site closest to their registered GP and the permanent site if they have mobility issues that mean climbing stairs is not possible or have additional needs.

### Intervention arm

Women registered at an intervention GP practice will receive the standard screening invitation letter at the usual planned time from the screening service with an additional approved information sheet included detailing instructions for booking the free door-to-door transport. This supplementary sheet will be included with the routine invitation letter posted to women by the BSS. Women are invited to attend the specific mobile unit or hospital site that is closest to their GP practice at the time of the initial invite. If participants in the intervention arm require a follow-up screening assessment, they can continue to use the free transport service.

All women in the intervention arm who attend the screening appointment will be asked to complete an intervention travel survey when they attend. 30 women in the intervention arm will be recruited for qualitative interviews to understand their experiences and perceptions of the door-to-door transport intervention.

### Control arm

Women at participating GP practices will receive the standard screening invitation letter at the usual planned time from the screening service. Women in the control arm who are attending their appointment will also be asked to complete a control arm travel survey when they attend their screening appointment.

### Eligibility criteria

Inclusion criteria:

- GPs located in ERY or Hull.
- Whose registered women meet the age criteria for the national breast screening programme (between 50 and 70 years old) and are due to be invited to attend a routine screening appointment during the intervention window.

Exclusion criteria:

- GP practices located outside of the set geographical area.
- Women with non-routine screening appointments.

### Randomisation

GP practices will be randomised 1:1 to the intervention arm or control arm with variable block sizes, stratified by area deprivation. Randomisation will be completed via the REDCap Cloud (RCC) online system provided by the Hull Health Trials Unit (HHTU).

### Sample size

The study sample size is estimated as 8000 women from eight GP practices. This is a pragmatic sample size based on the number of eligible women (between the ages of 50 and 70) at eight average sized GP practices. This sample size will allow for the reliable estimation of key feasibility figures and detection of signal of efficacy for the future full trial.^20^

### Data collection and statistical analysis

The feasibility outcomes, including recruitment figures, fidelity of intervention and outcome measure completion rates, will be reported and checked against the prespecified progression criteria. We will assess the screening uptake rates and report results by key characteristics (eg, ethnicity, area deprivation). The intraclass coefficient calculated from this data and effect size of intended outcomes will be used to inform the sample size (power calculation) for the definitive RCT. Participant data from the eight included GP practices collected by the HEY BSS will be linked to data held by NHS England (through the DARS process and following receiving Section 251 approval from Confidentiality Advisory Group (CAG) on deprivation scores for the residential locations and ethnicity data. Women who have opted out from the study or through the national opt-out will be removed from the data prior to it being sent to NHS England. Following linkage, NHS England will remove all patient identifiers and transfer the pseudonymised data to the University of Hull Data Safe Haven for analysis. The attendance data collected from HEY BSS for the study participants will be linked to this data set.

Data will be collected by the transport providers on trips taken to the breast screening appointments. Transport providers will be required to invoice for the cost of these journeys and will document the number of trips and duration (time) including waiting time between being dropped off for the appointment and being collected for the return journey. This cost per trip is an integral part of the costs that will be used in the cost-effectiveness analysis.

All participants, in both the control and intervention arms, will be invited to complete a travel survey on attending their screening appointment. The survey will gather detailed information on how participants travelled to the screening site, including mode of transport, journey duration, travel cost and whether they travelled alone. Participants will also be asked to provide their home postcode and starting location (eg, work or home), to enable estimation of travel distance and calculation of the study’s associated carbon footprint. The survey will further explore any difficulties encountered in attending the appointment, whether time had to be taken off work, and if the participant had previously attended a breast screening appointment. Additional demographic information will be collected, including GP practice, age group, ethnicity and whether English is their first language.

At the end of the travel survey, all women in the intervention arm will be asked whether they would be willing to take part in a follow-up interview for the qualitative component of the study. Those who express an interest will have the opportunity to provide their contact details within the survey for the research team to follow-up.

### Qualitative component

To explore women’s experiences and perceptions of the intervention, qualitative interviews will be conducted. In line with sample size recommendations for qualitative studies,^21^ we propose to interview approximately 30 women in the intervention arm. We will interview both women who attended and those who did not attend their screening appointments. Those who attended their screening appointments will be recruited through the final question on the travel survey. Women who did not attend their appointment (approximately 10) will be identified and contacted by HEY BSS.

The research team will recruit drivers (approximately 10) and transport provider managers to interview them about their views on the acceptability of the intervention.

The research team will send the volunteer participants an invitation letter, relevant participant information sheet detailing the purpose of the interview and what their participation would involve and a consent form. All the participants will complete a consent form before starting an interview. A copy of the study consent form is provided as online supplemental material (see separate uploaded document).

Interview recordings will be transcribed verbatim and analysed using thematic analysis.^22^ This would involve a three-stage process of identifying, analysing and interpreting themes (patterns of meanings) within the data. After data familiarisation, descriptive codes will be generated using words which describe participants’ views. These descriptive codes will be explored iteratively for patterns, similarities and differences, to develop themes. The themes will be reviewed in line with the research objectives and labelled accordingly. NVivo V.12 software (a qualitative data management software) will be used to manage the data analysis process. To ensure rigour, credibility and trustworthiness, data will be analysed by two researchers and conflicts resolved through discussion. A third researcher will be asked to mediate any unresolved conflict when necessary. Memos will also be kept to maintain an audit trail of the data analysis process. The theoretical framework of acceptability^23^ will provide a conceptual framework to understand the interview findings.

### Health economics

An economic evaluation will be conducted alongside the DOORSTEP feasibility trial. This will be conducted in two parts. Initially, a cost-effectiveness analysis will be conducted based on primary data collected within the trial. In addition, an economic model will be developed to explore the potential impact of the intervention on breast cancer detection and outcomes. Indicative estimates of cost-effectiveness will be generated. However, the key aim for the economic analysis in this feasibility study is to determine the plausibility of conducting an economic evaluation alongside a full trial, and to identify data gaps which would need to be addressed to facilitate this.

A decision analytical model will be developed in consultation with clinical experts, patient representatives and BSS, to facilitate exploration of the potential impact of the intervention on breast cancer detection and outcomes. The model will be generated using best practice methods, with the model structure, health states and parameter values being derived from the published literature, data from the DOORSTEP trial and where necessary, expert clinical opinion.^24^ A cost-utility analysis will be conducted based on the model outputs, following the National Institute for Health and Care Excellence (NICE) reference case for health technology appraisals.^25^ The outcome measure used in the model will be quality adjusted life years (QALYs) gained resulting from any increase in cancer detection as a result of screening. Results will be presented as expected incremental cost-effectiveness ratio which will be compared with the NICE cost-effectiveness threshold of £20 000–30 000 per QALY gained, and cost-effectiveness acceptability curves to demonstrate the probability of cost-effectiveness.^26^ Deterministic (one-way and scenario) and probabilistic (using Monte Carlo simulations) sensitivity analyses will be conducted to determine the impact of parameter value changes on estimates of cost-effectiveness and to establish the level of uncertainty surrounding the modelling results. A value of information analysis will also be conducted to estimate the value of conducting further research.^27^

The equity impact of the provision of free door-to-door transport for breast cancer screening will also be explored, for example, using distributional analysis or equity informative cost-effectiveness analysis (depending on data available), to identify potential distributional impacts on costs and benefits by measures of deprivation.^28^ This will be informed by area level deprivation data in relation to the location of the GP practice, along with data collected by the NHS BSS and NHS England.

### Outcome measures

This study is a feasibility trial with primary outcome measures designed to assess the feasibility of conducting a future definitive RCT. The primary and secondary feasibility outcomes are summarised below.

### Primary outcomes

The primary feasibility outcomes are summarised in table 1 along with criteria that will be used as a guide for progression to a future definitive phase III trial and described here:

1. Number of GP practices that agreed to participate and were randomised ahead of the scheduled breast screening invitation window.
2. Percentage of women invited from GP practices located in the 10% most deprived areas.
3. Percentage of intervention invites sent out by the BSS to all women during the scheduled breast screening invitation window.
4. Percentage of women who requested transport who were transported to and from their appointment by the service.
5. Acceptability of the intervention to the women and the service providers.
6. Completion and transfer of the screening and transport service data when required for analysis.

**Table 1:** Primary outcomes to assess feasibility and progression criteria

Feasibility outcomes	Outcome measure	Green	Amber	Red
Recruitment and randomisation of GP practices	Number of GP practices that agreed to participate and were randomised ahead of the scheduled breast screening invitation window.	8 (planned number of practices)	6–7 (numbers may still be achieved if invitations sent during part of the period, or less practices with more women)	<6 (insufficient number of practices/women included to provide data for sample size and feasibility estimates)
	Percentage of women invited from GP practices located in the 10% most deprived areas.	30%	15–30%	<15%
Fidelity of the transport intervention	Percentage of intervention invites sent out by the BSS to all women during the scheduled breast screening intervention window.	100% (all four intervention sites)	75%	<75%
Fulfilment of requests for the transport intervention	Percentage of women who requested transport who were transported to and from their appointment by the service.	100%	50–99% (explore reasons such as too many appts same date/time)	<50% (not viable to increase or change transport service to meet the demand)
Acceptability of the intervention to the women/service providers	Qualitative interviews.	Acceptable to all interviewed and no major barriers identified.	Acceptable with some modifications	Not acceptable to the majority of people interviewed
Completion and transfer of screening and transport service data when required for analysis	Screening service attendance data.	Screening data provided for all women in both arms of the trial.	Partial data provided	Data has not been transferred
	Transport providers.	Transport service data provided for all trips in the intervention arm of the trial.	Partial data provided	Data has not been transferred

BSS, breast screening service; GP, general practitioner.

### Secondary outcomes

The secondary outcomes are the intended outcomes for the definitive trial, and the data that will help inform sample size calculations for the phase III trial.

1. Screening uptake rates: percentage attendance at screening appointments. This will explore a potential signal of efficacy by comparing uptake between the transport intervention arm and the control arm. To provide context, these rates will be benchmarked against historical screening uptake from the previous 3 years for the included GP practices.
2. Transport intervention uptake rates.
3. Cost-effectiveness: reporting estimates of cost-effectiveness of the transport intervention.

## Data management

The main study database will be developed and managed by HHTU; University of Hull within the HHTU randomisation system (RCC). The database has been built and validated according to study-specific requirements. Study data will be recorded for both study administration and collection of participant data.

For the attendance data, HEY BSS will transfer participant data (including patient identifiers) directly to NHS England to link with ethnicity and deprivation data. After linkage, NHS England will transfer participant data with patient identifiers removed to the study team at the University of Hull (as pseudonymised data). This will be stored and analysed in the University of Hull Data Safe Haven. A participant notification poster will be displayed at participating GP practices throughout the trial to notify patients about the study and data linkage. A link will be provided to give them the option to opt out through the HEY BSS. All participants who have opted out will be removed by HEY BSS prior to transferring the data to NHS England.

The travel survey data will be collected by participants entering data directly into an online survey, or if preferred, they can complete paper forms and return to HHTU to be entered into the online survey by HHTU staff. The data will be collected according to the General Data Protection Regulation Act (2018). On completing the travel survey, the data will be held in the RCC where participants will be identified by a unique Subject ID Number.

Qualitative data sets which include identifiable information from interviews will be exported from the database periodically throughout the study. This data will be held in a secure area of BOX with access granted only to those who will be conducting interviews. Once interviews have been completed, personal data in the transcripts will be deleted.

Study data will be analysed by the HHTU statistician and research team. All data will be anonymised for the purposes of analysis and any subsequent reports or publications.

### Access to data

Direct access will be granted to authorised representatives from sponsor and the regulatory authorities to permit study-related monitoring, audits and inspections.

The database will be locked prior to export of the final data set. A copy of the final study data set and end of study notification will be sent to the sponsor by HHTU prior to the statistical analysis. A copy of the final study data set will be archived by the HHTU and sent to the chief investigator. Other authorised researchers requesting access to the data set for further research may apply through the chief investigator. Applications will be considered in keeping with the publications policy which will be agreed by the Trial Steering Committee (TSC).

### Patient and public involvement and engagement

The DOORSTEP study is supported by a public advisory group comprising five women of breast screening age. This group meets regularly to provide ongoing input and advice. Members of the group have played an active role in shaping the study from its inception, contributing to the development of participant-facing materials and reviewing the acceptability of the intervention. The group has been involved in discussions on all major aspects of the study design and implementation, including the use of NHS BSS data and the cost-effectiveness analysis. As the study progresses, they will review and sense-check the preliminary findings and support the dissemination of results to public audiences in accessible ways.

The Trial Management Group includes one public member who contributes to operational decisions, provides insight from a service-user perspective and acts as a link with the public advisory group.

There are also two public members representing women of breast screening age on the TSC, in an oversight and monitoring role.

All the public contributors involved in the study are supported in their role by a dedicated public involvement coordinator.

The research team is committed to ensuring meaningful public involvement at every stage of the study. This strengthens the relevance, transparency and accountability of the research and ensures that the perspectives of those most affected by breast screening policies remain central throughout the trial.

## Ethics and dissemination

### Regulatory approvals and trial oversight

The trial has been conducted in accordance with the principles of Good Clinical Practice in clinical trials, as applicable under UK regulations, the NHS Research Governance Framework.

The study protocol and amendments have been approved by London—Harrow Research Ethics Committee (REC reference: 24/LO/0037) on 19 February 2024. The study has been approved by the CAG (CAG reference: 4/CAG/0006) on 26 February 2024 and NHS England Screening Research, Innovation and Development Advisory Committee on 21 February 2024.

### Dissemination

Publications for the study will meet the standards required for submission to high quality peer reviewed journals. The results will be disseminated in peer-reviewed journals, through local and other relevant clinical networks and at national and international meetings. Participants will be sent a summary of the findings, if requested, and a copy of the final accepted manuscript of the primary paper after the results have been published.

## Protocol version

This paper is based on Protocol V.1.1 20.JAN.2024.

## Current trial status

The DOORSTEP study opened to recruitment in August 2024 and has a 12-month recruitment window.

## Supplementary material

10.1136/bmjopen-2025-108616 online supplemental file 1
