# Supplementary material for: Evaluating the effectiveness and acceptability of free door-to-door transport to increase the uptake of breast screening appointments in Yorkshire: a cluster randomised GP feasibility trial (DOORSTEP protocol)
Source: BMJ Open. 2026 Jan 28;16(1):e108616. doi: 10.1136/bmjopen-2025-108616 (PMC12853533; doi:10.1136/bmjopen-2025-108616)
Supplement: online supplemental file 1 [file bmjopen-16-1-s001.pdf]

Evaluating the effectiveness and acceptability of free door to door transport to increase the uptake of breast screening appointments in Yorkshire

INFORMED CONSENT FORM

Name of Researcher: ___________________________

Subject ID Number: __ __ __ - __ __ - __ __ ***[PAT/TRA] – [GP code] – [Subject ID]***

**This form will be kept confidential and not released to anyone outside the research team**		***Please initial each box below***	
**1**	I confirm that I have read and understand the Information Sheet [Version x.x dated xx/xx/xxxx] for the above study and have had the opportunity to consider the information, ask questions and have had these answered satisfactorily.		
**2**	I understand that my participation is voluntary and that I am free to withdraw at any time without giving any reason and without my medical care or legal rights being affected.		
**3**	I understand that if I withdraw at any point during the interview, any information recorded up to that point will be retained and used in the study.		
**4**	I agree for the interview to be audio-recorded, and sent for transcribing by a member of the study team.		
**5**	I understand that my words may be used as anonymised quotes in reports of the study published in academic journals or presented at conferences but that I will not be directly identified (e.g. that my name will not be used).		
**6**	I understand that relevant sections of any of the study data may be looked at by responsible individuals from the research team, University of Hull, relevant third parties or from regulatory authorities where it is relevant to my taking part in the research. I give permission for these individuals to access my records.		
**7**	I consent to the secure transfer, storage and use of paper and electronic personal information, for the purposes of this study to the Hull Health Trials Unit, or relevant third parties. I understand that any information that could identify me will be kept strictly confidential and that no personal information will be included in the study report or other publication.		
**8**	I agree to take part in this research project and agree for my data to be used for the purpose of this study and in future research.		
**9**	I would like to receive a summary of the findings of this study when it is complete.	YES	
		NO	

INFORMED CONSENT FORM

Evaluating the effectiveness and acceptability of free door to door transport to increase the uptake of breast screening appointments in Yorkshire

Subject ID Number: __ __ __ - __ __ - __ __ ***[PAT/TRA] – [GP code] – [Subject ID]***

Name of participant (*please print*)	Date	Signature of participant

Name of person receiving consent (*please print*)	Date	Signature of person receiving consent

1 copy for participant; 1 original for Investigator Site File;
Upload 1 copy onto the study database for HHTU
