# Supplementary material for: Evaluating the effectiveness and acceptability of free door-to-door transport to increase the uptake of breast screening appointments in Yorkshire: a cluster randomised GP feasibility trial (DOORSTEP protocol)
Source: BMJ Open. 2026 Jan 28;16(1):e108616. doi: 10.1136/bmjopen-2025-108616 (PMC12853533; doi:10.1136/bmjopen-2025-108616)
Supplement: Supplementary file 2 [file bmjopen-2025-108616.reviewer_comments.pdf]

PEER REVIEW HISTORY

BMJ Open publishes all reviews undertaken for accepted manuscripts. Reviewers are asked to complete a checklist review form and are provided with free text boxes to elaborate on their assessment. These free text comments are reproduced below.

ARTICLE DETAILS

Title (Provisional)

Evaluating the effectiveness and acceptability of free door to door transport to increase the uptake of breast screening appointments in Yorkshire: A cluster randomised GP feasibility trial (DOORSTEP protocol)

Authors

Haji Sadeghi, Mahboobeh; Roberts1, Helen; Acaster1, Beccy; Miles1, Hannah; Pitel1, Lukas; Abass2, Wessam; Peacock3, Lesley; Kelly, Charlotte; Cohen, Judith; Bamidele, Olufikayo; Williams, Bronwen; Huang, Chao; Dawkins, Bryony; Macleod, Una

---

VERSION 1 - REVIEW

**Reviewer**	**1**
**Name**	**Neven, P.**
**Affiliation**	**Univ Hosp Leuven, Multidiscplinary Breast Center**
**Date**	**21-Oct-2025**
**COI**	**None**

This is a nice study to see how participation at breast cancer screening can be improved offering door-to-door transport.

The study sample size is estimated as 8000 women from eight GP practices. This is a

pragmatic sample size based on the number of eligible women (between the ages of 50 and 70) at eight average sized GP practices.

The research question is not clear to me (and a bit obscure). Any outcome to be of clinical meaningfullness will depend on the more specific 'main' study objective(s) and secondary endpoints still to be defined. Including 8000 women (1x per 3 year breast cancer screening) with an already 72% participation rate might not be enough. How will they take prior mammogram results intoaccount or do they all have a first ever mammogram in their life? The study is already ongoing but will recruit for how long and how many screening rounds?

Why a bit obscure? This sample size will allow for the reliable estimation of key feasibility figures and detection of signal of efficacy for the future full trial.--> this is not very clear to me what exactly will they study beyond getting more patients aware of screening and fascilitating transport?

How do they differentiate the different and well know -very heterogeneous- reasons not to participate at a previous round (for those already refusing one or 2 rounds?). Also, some of the participants might have undergo high risk screening images or opportunistic screening within the range of the previous round. do the investigators take these potential risks -regarding compliance to breast cancer screening- into account?

Please define when this study can be considered as 'positive' for other health communities within the UK and Europe.

**Reviewer**	**2**
**Name**	**Jones, Lyn**
**Affiliation**	**North Bristol NHS Trust, Bristol Breast Care Centre**
**Date**	**26-Oct-2025**
**COI**	**None**

Manuscript Summary

The manuscript describes a study protocol to investigate the feasibility of providing free door to door transport to increase breast screening uptake, using a cluster randomised design of GP practices in Yorkshire. The authors represent a collaboration between the Universities of Hull (study sponsor) and of Leeds, including authors from the Hull Health Trials Unit.

Major points

Dates of the study

1. Protocol papers should report planned or ongoing studies and the study dates should be included in the manuscript. On the ISRCTN webpage for the study, it states that the first enrolment in the study was 12/6/2024 and the final enrolment was 01/03/2025. The study completion date is listed as 31/01/2026, just over 3 months from now. Please could the authors clarify if these dates are correct and whether data collection has yet completed and whether data analysis has yet begun?

2. Under the heading, “current trial status”, the manuscript states that the DOORSTEP study opened to recruitment in August 2024 and has a 12-month recruitment window (page 10, lines 3-7). This is inconsistent with the trial dates given on the study’s ISRCTN webpage (see point 1 above).

General comments

The manuscript appears well written and is likely to be of interest to the BMJ Open readership. To my knowledge, the research it describes is novel and the study has been approved by the NHS Breast Screening Programme Research Innovation and Development Advisory Committee (RIDAC), appropriately.

Specific comments

Title

The title adequately states the trial design, population and interventions and it identifies the manuscript as a study protocol.

Abstract

The abstract is appropriately structured and complete in accordance with the SPIRIT checklist.

Introduction

The Introduction section adequately explains the background to and the justification for undertaking the study.

Methods and analysis

3. The instructions to authors within the included SPIRIT checklist require mention in the methods and analysis section that the SPIRIT reporting guidelines were used in the reporting of this study protocol (with the appropriate additional reference). I could neither find mention of the SPIRIT checklist in the Methods and Analysis section of the manuscript, nor a reference for the SPIRIT checklist among the manuscript references.

4. Study aims and objectives (page 3, lines 18-37): The study aims and objectives are currently written as a paragraph of prose which makes the individual objectives difficult to appreciate at a glance. Additionally, the text includes justification of the research which is not relevant to this study heading. Replacing the prose with an initial statement of the study aim(s) followed by a list of the study objectives as bullet points would improve the clarity of this section.

5. The authors state in the methods and analysis section (page 4, lines 31-35 and page 5, lines31-35) that 8 average sized GP practices is estimated to yield 8000 participants (women aged 50-70), but the reference given does not explain the basis for this (evidence for GP practice size and numbers of women by age). Please could an appropriate reference be additionally provided?

6. Also relevant to the previous point, in Table 1 (page 15), the authors present the pre-defined progression criteria for the feasibility trial to progress to a definitive trial of the intervention. The first of the listed outcome measures is the number of GP practices agreeing to participate and being randomised ahead of the scheduled breast screening invitation window, but there is no indication of the denominator for this measure, namely how many

GP practices are likely to be eligible for inclusion in the study (located in ERY or Hull and whose registered women are due to be invited to attend a routine screening appointment during the intervention window). For example, does this progression criterion ask for 8 of 10 practices or for 8 of 20 practices? Without this information it is difficult to fully appreciate the value of this progression criterion.

7. In the Secondary Outcomes section (page 8, lines 7-14), Screening uptake rates is the first to be listed for exploration as a comparator for the future full trial, between the intervention arm and the control arm. Will this be presented in the context of screening uptake rates for previous years for the included GP practices (for both intervention and control arms)? If not, what is the justification for not including this context within a feasibility study where numbers in the two groups will be underpowered to report a significant difference between them?

8. The authors state that the travel survey will collect self-reported data on whether participants have previously attended a screening appointment (page 7, lines 11&12). Will more detailed information on the breast screening invitation type (held by the screening service) be included in the analysis (other than that self-reported by participants in the travel survey)? Breast screening uptake in the NHS breast screening programme varies by invitation type, age, geographic region socio-economic status and ethnicity, so the invitation type if not reliably collected from source could act as a significant confounder (reference: Breast screening programme England 2022-2023 from NHS Digital: section 4, by invitation type, available at: https://digital.nhs.uk/data-and-information/publications/statistical/breast-screening-programme/england---2022-23/mainreport2223). For example, a graph within this publication quotes an uptake rate of 81.1% for "routine invitation (last screen within 5 years)" and 41.4% for "routine invitation (last screen more than 5 years)". Additionally, a quotation from this publication that demonstrates the wide variation in uptake of screening dependant on screening invitation type is as follows:

• "Uptake was lowest (14.3%) amongst women who received a routine invitation, having failed to respond to a previous invitation(s).

• Uptake was highest (98.8%) amongst those receiving short term recall invitations."

9. It would be of interest to readers for additional information to be included about the mechanism by which the intervention (information about booking free door-to-door transport) will be added to the routine invitation to screen letters for eligible women in the GP practices randomised to receive the invitation.

Ethics and Dissemination

10. Please could the dates of the regulatory approvals being granted (REC, CAG and RIDAC) be included in the section on Regulatory approvals and trial oversight (page 9, lines43-47)?

Tables and figures

The table and figure included with the manuscript are clear and add to the understanding of the manuscript for the reader.

**Reviewer**	**3**
**Name**	**Lofters, Aisha**
**Affiliation**	**University of Toronto, Department of Family and Community Medicine**
**Date**	**03-Nov-2025**
**COI**	**None**

Thanks for the opportunity to review this study protocol for a feasibility trial on breast cancer screening transport. Please see some comments below for further details that would be helpful for readers:

INTRODUCTION

A brief bit of context on the jurisdiction of Yorkshire would be helpful for international readers.

STUDY DESIGN

For women who need follow-up (and may have a cancer diagnosed), at what point does the free transport stop? Or would the diagnostic phases occur at a different location?

STUDY POPULATION

Does the estimated sample size take into account the number of women at each GP practice who will not actually be due for screening in the 12-month recruitment period?

STUDY RECRUITMENT

Can you share a bit more about the mobile vans and where they go? How do people know where the vans will be and when?

OUTCOMES

I would consider documenting the number of GP practices approached to get to the number that eventually agree. Will screening uptake rates also be looked at by sociodemographic characteristics?

PUBLIC ADVISORY GROUP

Are women of low SES represented among the 5, and if not, how has that patient voice been incorporated?

VERSION 1 - AUTHOR RESPONSE

Reviewer: 1

Dr. P. Neven, Univ Hosp Leuven

Comments to the Author:

This is a nice study to see how participation at breast cancer screening can be improved offering door-to-door transport.

Thank you for your comment.

The study sample size is estimated as 8000 women from eight GP practices. This is a pragmatic sample size based on the number of eligible women (between the ages of 50 and 70) at eight average sized GP practices.

Yes, that is correct.

The **research question** is not clear to me (and a bit obscure). Any outcome to be of clinical meaningfulness will depend on the more specific 'main' study objective(s) and secondary endpoints still to be defined. Including 8000 women (1x per 3 year breast cancer screening) with an already 72% participation rate might not be enough. How will they take prior mammogram results into account or do they all have a first ever mammogram in their life? The study is already ongoing but will recruit for how long and how many screening rounds?

Why a bit obscure? This sample size will allow for the reliable estimation of key feasibility figures and detection of signal of efficacy for the future full trial.--> this is not very clear to me what exactly will they study beyond getting more patients aware of screening and facilitating transport?

Thank you for your thoughtful comments. We appreciate your observation that the research question appeared unclear or somewhat obscure. We have revised the manuscript to more explicitly articulate the aims of the feasibility study.
To clarify, this feasibility study is not designed to evaluate clinical effectiveness. Rather, the sample size is intended to provide reliable estimates of key feasibility parameters—including recruitment rates, acceptability of the intervention, retention, and completeness of data collection—as well as to detect any early signals that may inform the design of a future full-scale trial. We have made this purpose clearer in the manuscript by adding Table 1 Primary Outcomes to assess feasibility and progression criteria in Primary Outcomes section page 8.

The primary aim of this feasibility study is to assess the practicality of the trial procedures, recruitment strategies, and data-collection processes, rather than to measure statistically significant differences between intervention and control groups. Although we will compare screening uptake rates as an exploratory secondary outcome, these results are intended only to guide refinement of the intervention and inform sample size calculations for the definitive trial.

We appreciate the reviewer's insightful comment. Screening uptake rates will indeed be compared between the intervention and control arms as part of the exploratory secondary outcomes. However, we do not intend to present these findings in the context of historical screening uptake rates from previous years for the included GP practices.

How do they differentiate the different and well know -very heterogeneous- reasons not to

participate at a previous round (for those already refusing one or 2 rounds?). Also, some of the participants might have undergo high risk screening images or opportunistic screening within the range of the previous round. do the investigators take these potential risks -regarding compliance to breast cancer screening- into account?

Thank you for your comment. This is an interesting issue, but it falls outside the remit of this trial. The intervention is administrated by the breast screening service and the intervention letter going to everybody in the intervention arm that is being invited for routing screening. We do not have any information of different risks of previous rounds of screening for these women.

Please define when this study can be considered as 'positive' for other health communities within the UK and Europe.

This is a feasibility trial, and we are currently applying for the full trial. This feasibility trial will give a signal of efficacy.

Reviewer: 2

Dr. Lyn Jones, North Bristol NHS Trust

Comments to the Author:

Manuscript Summary

The manuscript describes a study protocol to investigate the feasibility of providing free door to door transport to increase breast screening uptake, using a cluster randomised design of GP practices in Yorkshire. The authors represent a collaboration between the Universities of Hull (study sponsor) and of Leeds, including authors from the Hull Health Trials Unit.

Thank you for your comment, that is correct.

Major points

Dates of the study

1. Protocol papers should report planned or ongoing studies and the study dates should be included in the manuscript. On the ISRCTN webpage for the study, it states that the first enrolment in the study was 12/6/2024 and the final enrolment was 01/03/2025. The study completion date is listed as 31/01/2026, just over 3 months from now. Please could the authors clarify if these dates are correct and whether data collection has yet completed and whether data analysis has yet begun?

Thank you for your comment the IRSCTN now updated as below;
The final enrolment will be on 30/11/2025.
We may extend the study end data, but current date stated in the protocol paper is correct.

2. Under the heading, "current trial status", the manuscript states that the DOORSTEP study opened to recruitment in August 2024 and has a 12-month recruitment window (page 10, lines 3-7). This is inconsistent with the trial dates given on the study's ISRCTN webpage (see point 1 above).

Thank you for your comment the IRSCTN now updated General

comments

The manuscript appears well written and is likely to be of interest to the BMJ Open readership. To my knowledge, the research it describes is novel and the study has been approved by the NHS Breast Screening Programme Research Innovation and Development Advisory Committee (RIDAC), appropriately.

Thank you for your comment.

Specific comments

Title

The title adequately states the trial design, population and interventions and it identifies the manuscript as a study protocol.

Thank you for your comment.

Abstract

The abstract is appropriately structured and complete in accordance with the SPIRIT checklist.

Thank you for your comment.

Introduction

The Introduction section adequately explains the background to and the justification for undertaking the study.

Thank you for your comment.

Methods and analysis

3. The instructions to authors within the included SPIRIT checklist require mention in the methods and analysis section that the SPIRIT reporting guidelines were used in the reporting of this study protocol (with the appropriate additional reference). I could neither find mention of the SPIRIT checklist in the Methods and Analysis section of the manuscript, nor a reference for the SPIRIT checklist among the manuscript references.

Thank you for your comment. We can confirm that we completed the SPIRIT checklist and submitted it with the manuscript. We reported our feasibility trial inline with the SPIRIT guidelines. We have revised the methods and analysis section to state that the SPIRIT guidelines were followed and added the appropriate SPIRIT referenced on page 3.

"This protocol was prepared in accordance with the SPIRIT (Standard Protocol Items: Recommendations for Interventional Trials) 2013 reporting guidelines (18)."

4. Study aims and objectives (page 3, lines 18-37): The study aims and objectives are currently written as a paragraph of prose which makes the individual objectives difficult to appreciate at a

glance. Additionally, the text includes justification of the research which is not relevant to this study heading. Replacing the prose with an initial statement of the study aim(s) followed by a list of the study objectives as bullet points would improve the clarity of this section.

Thank you for your comment. We agree that presenting the aims and objectives as bullet points would improve clarity. We have revised this section accordingly by providing a clear statement of the study aim followed by a bullet-point list of the individual objectives, and we have removed the justification text from this heading.

5. The authors state in the methods and analysis section (page 4, lines 31-35 and page 5, lines31-35) that 8 average sized GP practices is estimated to yield 8000 participants (women aged 50-70), but the reference given does not explain the basis for this (evidence for GP practice size and numbers of women by age). Please could an appropriate reference be additionally provided?

Thank you for your comment. The estimate of approximately 8,000 eligible women is based on internal data provided by the local breast screening service for the region in which the study is being conducted. As these figures come from service-level operational data rather than published sources, an external reference is not available. We have clarified this in the manuscript. Reference 20 refers to the justification of 8 GP practices.

6. Also relevant to the previous point, in Table 1 (page 15), the authors present the pre-defined progression criteria for the feasibility trial to progress to a definitive trial of the intervention. The first of the listed outcome measures is the number of GP practices agreeing to participate and being randomised ahead of the scheduled breast screening invitation window, but there is no indication of the denominator for this measure, namely how many GP practices are likely to be eligible for inclusion in the study (located in ERY or Hull and whose registered women are due to be invited to attend a routine screening appointment during the intervention window). For example, does this progression criterion ask for 8 of 10 practices or for 8 of 20 practices? Without this information it is difficult to fully appreciate the value of this progression criterion.

Thank you for your comment. The progression criterion specifies that we aim to recruit 8 GP practices. The feasibility aim is to assess whether we can achieve this target within the scheduled invitation window. The exact number of eligible practices in the region is not fixed, as this can change over time due to mergers or other organizational changes. We will report the number of GP practices successfully recruited when presenting the study results.

We have also included a reference on page 5 (reference 20) for the statistical approach used to define this progression criterion.

7. In the Secondary Outcomes section (page 8, lines 7-14), Screening uptake rates is the first to be listed for exploration as a comparator for the future full trial, between the intervention arm and the control arm. Will this be presented in the context of screening uptake rates for previous years for the included GP practices (for both intervention and control arms)? If not, what is the justification for not including this context within a feasibility study where numbers in the two groups will be underpowered to report a significant difference between them?

Thank you for your comment. While the main outcomes of this feasibility study relate to feasibility measures, we will report screening uptake rates between the intervention and control

arms as a signal of potential efficacy. To provide context, we will benchmark these rates against historical screening uptake from the previous three years for the included GP practices. This will allow us to interpret the findings in relation to previous screening patterns, even though the study is not powered to detect statistically significant differences between groups.

8. The authors state that the travel survey will collect self-reported data on whether participants have previously attended a screening appointment (page 7, lines 11C12). Will more detailed information on the breast screening invitation type (held by the screening service) be included in the analysis (other than that self-reported by participants in the travel survey)? Breast screening uptake in the NHS breast screening programme varies by invitation type, age, geographic region socio-economic status and ethnicity, so the invitation type if not reliably collected from source could act as a significant confounder (reference: Breast screening programme England 2022-2023 from NHS Digital: section 4, by invitation type, available at: https://digital.nhs.uk/data-and-information/publications/statistical/breast-screening-programme/england---2022-23/mainreport2223). For example, a graph within this publication quotes an uptake rate of 81.1% for "routine invitation (last screen within 5 years)" and 41.4% for "routine invitation (last screen more than 5 years)". Additionally, a quotation from this publication that demonstrates the wide variation in uptake of screening dependant on screening invitation type is as follows:

• "Uptake was lowest (14.3%) amongst women who received a routine invitation, having failed to respond to a previous invitation(s).

• Uptake was highest (98.8%) amongst those receiving short term recall invitations."

Thank you for your comment. The travel survey collects self-reported information on whether participants have previously attended a screening appointment. However, we are not collecting detailed individual-level data on previous attendance from the screening service. We are focusing on overall uptake at the GP level. However we'll be reporting results by age, deprivation and ethnicity to look at differences by groups.

9. It would be of interest to readers for additional information to be included about the mechanism by which the intervention (information about booking free door-to-door transport) will be added to the routine invitation to screen letters for eligible women in the GP practices randomised to receive the invitation.

Thank you for your comment. We have provided additional information in the manuscript on page 5 to clarify that a separate sheet of paper detailing the free door-to-door transport offer is included alongside the routine screening invitation letters for eligible women in the GP practices randomized to receive the intervention.

Ethics and Dissemination

10. Please could **the dates of the regulatory approvals** being granted (REC, CAG and RIDAC) be included in the section on Regulatory approvals and trial oversight (page 9, lines43-47)?

Thank you for your comment, Ethics and Dissemination was amended as your request.

Tables and figures

The table and figure included with the manuscript are clear and add to the understanding of the manuscript for the reader.

Thank you for your comment.

Reviewer: 3

Dr. Aisha Lofters, University of Toronto, St Michael's Hospital, Women's College Hospital, ICES, University of Toronto, Women's College Hospital, Dalla Lana School of Public Health

Comments to the Author:

Thanks for the opportunity to review this study protocol for a feasibility trial on breast cancer screening transport. Please see some comments below for further details that would be helpful for readers:

INTRODUCTION

A brief bit of context on the jurisdiction of **Yorkshire** would be helpful for international readers.

Thank you for your comment. We have added a short description of the Yorkshire region on page 2 to provide context for international readers.

STUDY DESIGN

For women who need follow-up (and may have a cancer diagnosed), at what point does the free transport stop? Or would the diagnostic phases occur at a different location?

Thank you for your comment. The free transport offer applies to follow-up appointments at any location, this was added on page 5 intervention arm section.

STUDY POPULATION

Does the estimated sample size take into account the number of women at each GP practice who will not actually be due for screening in the 12-month recruitment period?

Thank you for your comment. All women within a GP practice are invited for screening at the same time, and invitations occur every three years. The estimated sample size reflects the number of women expected to be invited during the recruitment period.

STUDY RECRUITMENT

Can you share a bit more about the mobile vans and where they go? How do people know where the vans will be and when?

Thank you for your comment. We have reviewed section Intervention Arm page 5 and added more on the process.

"This supplementary sheet will be included with the routine invitation letter posted to women by the breast screening service. Women are invited to attend the specific mobile unit or hospital site that is closest to their GP practice at the time of the initial invite. If participants in the

intervention arm require a follow up screening assessment, they can continue to use the free transport service."

OUTCOMES

I would consider documenting the number of GP practices approached to get to the number that eventually agree. Will screening uptake rates also be looked at by sociodemographic characteristics?

Thank you for your comment, we will be documenting this as part of our records. We'll be reporting results by age, deprivation and ethnicity to look at differences by groups.

PUBLIC ADVISORY GROUP

Are women of low SES represented among the 5, and if not, how has that patient voice been incorporated?

Thank you for your comment. Yes, two of the five women are from lower socioeconomic backgrounds. The Ethics Committee provided positive feedback on the representation and input of the Public Advisory Group.

VERSION 2 - REVIEW

**Reviewer**	**1**
**Name**	**Neven, P.**
**Affiliation**	**Univ Hosp Leuven, Multidiscplinary Breast Center**
**Date**	**26-Nov-2025**
**COI**	

---

they did take comments into account

**Reviewer**	**2**
**Name**	**Jones, Lyn**
**Affiliation**	**North Bristol NHS Trust, Bristol Breast Care Centre**
**Date**	**09-Dec-2025**
**COI**	

Thank you for addressing my previous comments. Wishing you well for the study and looking forward to seeing the results.
